# Granulocyte colony-stimulating factor enhances the therapeutic efficacy of bone marrow mesenchymal stem cell transplantation in rats with experimental acute pancreatitis

**DOI:** 10.18632/oncotarget.15515

**Published:** 2017-02-19

**Authors:** Bo Qu, Yanjie Chu, Fang Zhu, Beibei Wang, Ting Liu, Bo Yu, Shizhu Jin

**Affiliations:** ^1^ Department of Gastroenterology and Hepatology, The Second Affiliated Hospital, Harbin Medical University, Harbin, 150086, China; ^2^ Department of Gastroenterology and Hepatology, The First People's Hospital of Yongkang, Zhejiang Province, 321300, China; ^3^ Department of the Second Internal Medicine Ward, The Yellow River Hospital, Tianjin, 300101, China; ^4^ Department of Gastroenterology and Hepatology, The Tailai County People's Hospital, Heilongjiang Province, 162400, China

**Keywords:** bone marrow stem cell transplant, granulocyte colony-stimulating factor, acute pancreatitis, transdifferentiation

## Abstract

**Introduction:**

Acute pancreatitis (AP) is one of the most common diseases involving necrotic inflammation. Bone marrow mesenchymal stem cells (BMMSCs) have the potential of multi-directional differentiation and self-renewal for tissue repair. It remains less clear if granulocyte colony-stimulating factor (G-CSF) can improve the therapeutic effect of BMMSC transplant in AP. Therefore, we explored this issue in a rat model of experimental AP.

**Results:**

Transplanted PKH26-positive BMMSCs were present in the injured pancreatic tissue, with some cells co-expressed pancreatic cellular markers, including Pax-4, Ngn3 and Nkx-6. Pathological, biochemical and serological data suggested an improvement in histological and functional recovery in these animals relative to control. Overall, the AP model rats received BMMSCs and G-CSF co-treatment showed better recovery in terms of tissue regeneration and blood biochemical levels relative to other groups.

**Materials and Methods:**

BMMSCs from donor rats were labeled with the fluorescent dye PKH26 and transfused into recipient rats with AP induced by L-arginine. The animals were divided into a control group, and groups treated with BMMSCs, G-CSF, and BMMSCs together with G-CSF. Therapeutic effects were evaluated histologically with immunohistochemistry and immunofluorescence, together with biochemical measurement of pancreatic markers.

**Conclusion:**

G-CSF therapy with BMMSC transplantation improves histological and functional outcomes in rats with experimental AP.

## INTRODUCTION

Acute pancreatitis (AP) is a common necrotizing inflammatory disease. AP patients have unbearable abdominal pain, abdominal distention, and in severe cases, concurrent systemic organ failure leading to high the mortality [1]. AP is characterized by inflammation with the activation of digestive proenzymes, including proteases, lipase, and amylase, that results in autodigestive destruction of the pancreatic parenchyma and the peripancreatic adipose tissue [2]. Additionally, AP is associated with pancreatic edema, hemorrhage and necrosis [3, 4]. Its pathogenic mechanism is still not very clear, while a common view is that the interaction of proinflammatory cytokines such as interleukin (IL)-1, tumor necrosis factor (TNF), IL-6, IL-8 and IL-4 with components of the anti-inflammatory response system, such as IL-10, causes severe complications of AP including pancreatic necrosis [5].

Despite various experimental and clinical tests of potential drugs, only a few pharmacologic options are currently available for the treatment of AP [6]. In recent years, stem cell therapy for AP has emerged as a new option [7–9]. Mesenchymal stem cells (MSCs) have the potential to differentiate into bone, cartilage, or adipose tissue under suitable conditions [10, 11]. These non-hematopoietic stromal progenitor cells have self-renewal ability and high plasticity. Evidence suggests that bone marrow mesenchymal stem cells (BMMSCs) can differentiate into pancreatic stem cells, which may mitigate pancreatic injury [12–17]. However, it appears that the effect of BMMSC therapy may be not sufficient as, for instance, the amount of transplanted BMMSCs is limited, or inflammatory mediators affect the survival and differentiation of the cells after transplantation [18]. Granulocyte colony-stimulating factor (G-CSF) can mobilize BMMSCs *in vivo* and has been used in the treatment of neutropenia and for bone marrow reconstitution and stem cell mobilization [19, 20]. The multi-directional differentiation potential of BMMSCs and the mobilization effect of G-CSF form a tangible theoretical basis for the treatment of AP. Therefore, we attempted to explore a synergetic therapeutic benefit of combining BMMSC transplantation and G-CSF administration in a rat model of AP.

## RESULTS

### Evaluation of L-arginine induced pancreatic injury

**Figure 1:**
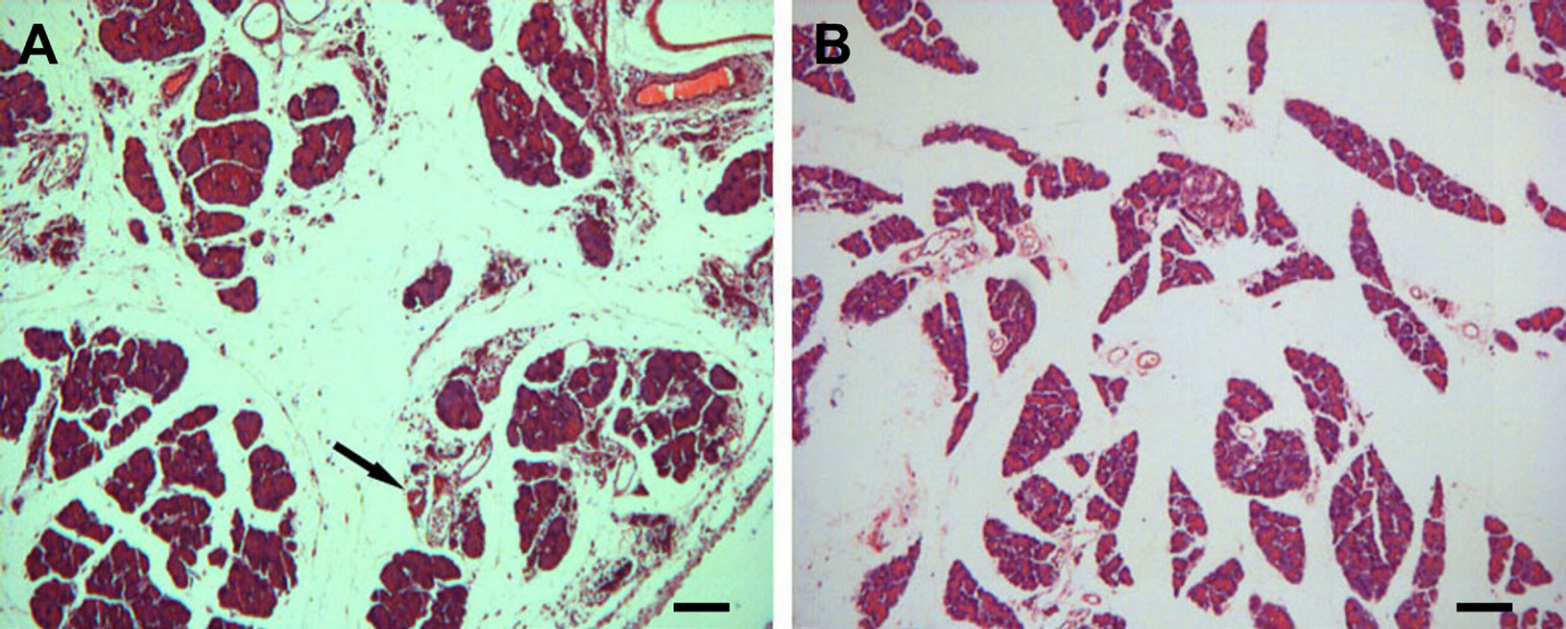
Histological evaluation of pancreatic lesions (**A**) after L-arginine administration with hematoxylin and eosin (H.E) stain relative to normal control (**B**) (10×). (A) In the normal rats, the microscopic structure of the pancreas is clear; the glandular lobule is complete, without interstitial leakage. (B) Injured pancreas shows interstitial edema, inflammatory cell infiltration, and necrosis. Scale bar = 100 μm.

On microscopic examination, the structure of the pancreas was intact in the normal rats, showing complete glandular and lobular organization and distinct pancreatic cytology, without interstitial leakage (Figure 1A). In contrast, in H.E. preparation of the pancreas from the AP model rats, there was apparent structural disruption, with large areas of the tissue showing necrosis, hemorrhage and inflammatory cell infiltration (Figure 1B).

### Characterization of cultured BMMSCs *in vitro*

**Figure 2:**
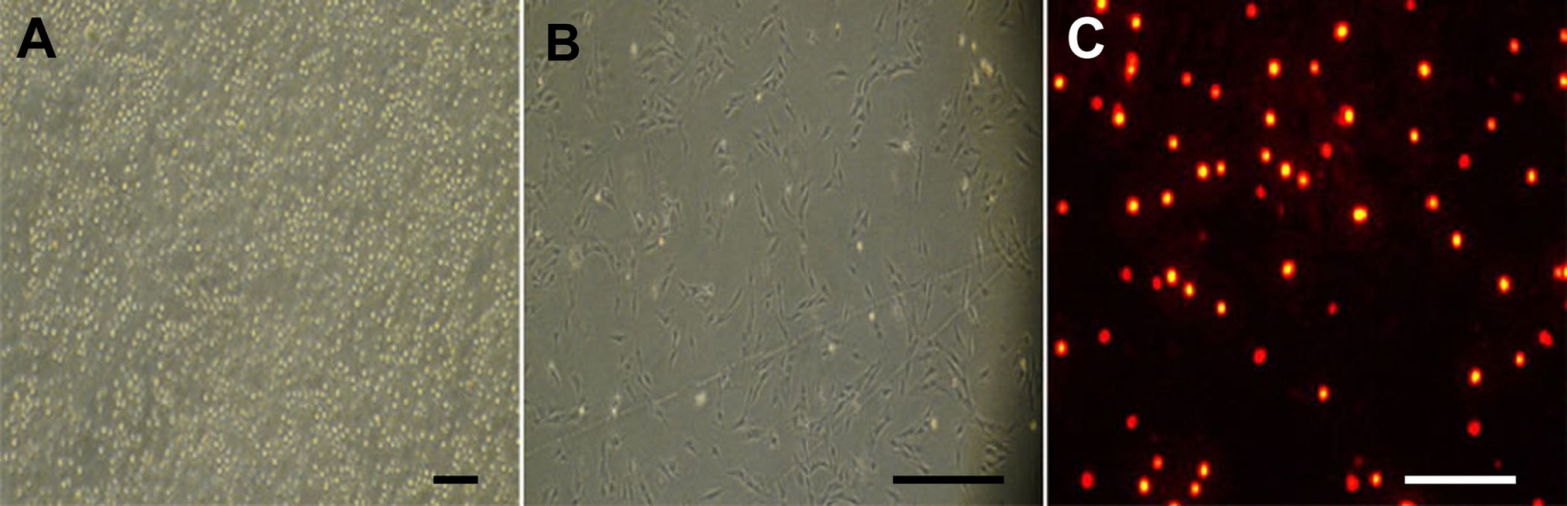
Morphological feature of cultured bone marrow mesenchymal stem cells (BMMSCs) harvested from donor rats (**A**) On the first day cultured cells largely remained floating with a few cells adherent to bottle wall. (**B**) On the fifth day, a large number of spindle cells were attached to the bottle wall. (**C**) The cells were tagged with the red fluorescent dye PKH26 after 2 weeks. Scale bar = 100 μm.

On the first day of culture, a small amount of cells stuck to the bottom of the bottle, while the majority remained floating in the medium (Figure 2A). A large amount of spindle cells were seen at the bottom on the fifth day (Figure 2B). Following PKH26 labeling, a great amount of cells exhibited bright red fluorescence *in vitro* for 2 weeks (Figure 2C).

**Figure 3:**
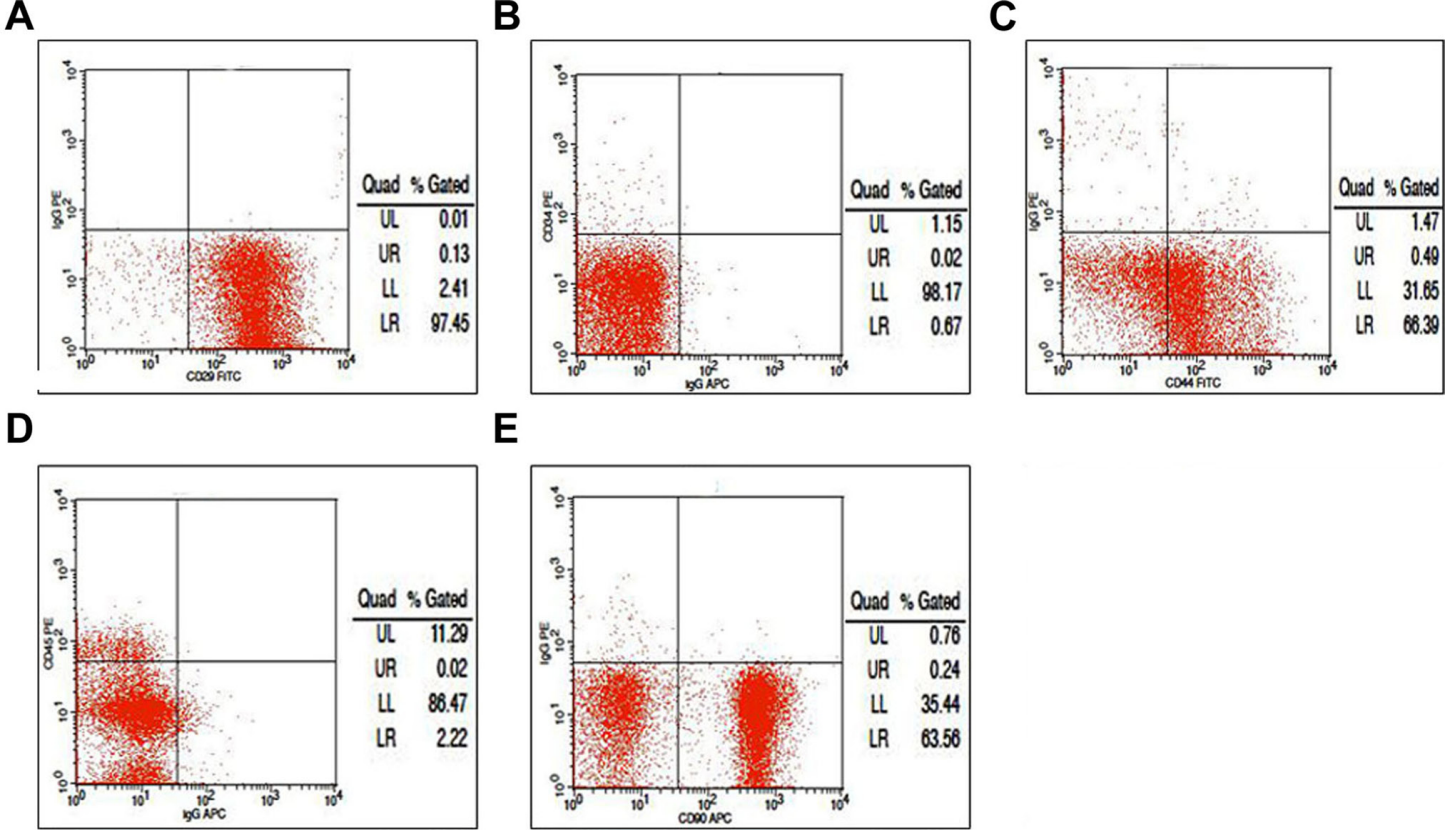
Fluorescence-activated cell sorting analysis with markers of bone marrow mesenchymal stem cells (BMMSCs) (**A**) We found that 97.58% of the isolated cells expressed CD29; (**B**) 0.69% expressed CD34; (**C**) 66.88% expressed CD44; (**D**) 2.24% expressed CD45; and (**E**) 63.80% expressed CD90.

FACS assays confirmed that 97.58% of the isolated cells expressed CD29 (Figure 3A), 0.69% expressed CD34 (a myeloid progenitor cell antigen also presented in endothelial cells and some fibroblasts, Figure 3B); 66.88% expressed CD44 (a stem cell marker, Figure 3C), 2.24% expressed CD45 (a hematopoietic and leukocyte marker, Figure 3D) [21]; and 63.80% of the cells expressed CD90 (Figure 3E). Thus, the cells showed the antigenic profile of BMMSCs [22].

### Colocalization of transplanted BMMSCs in the pancreas of recipient rats

**Figure 4:**
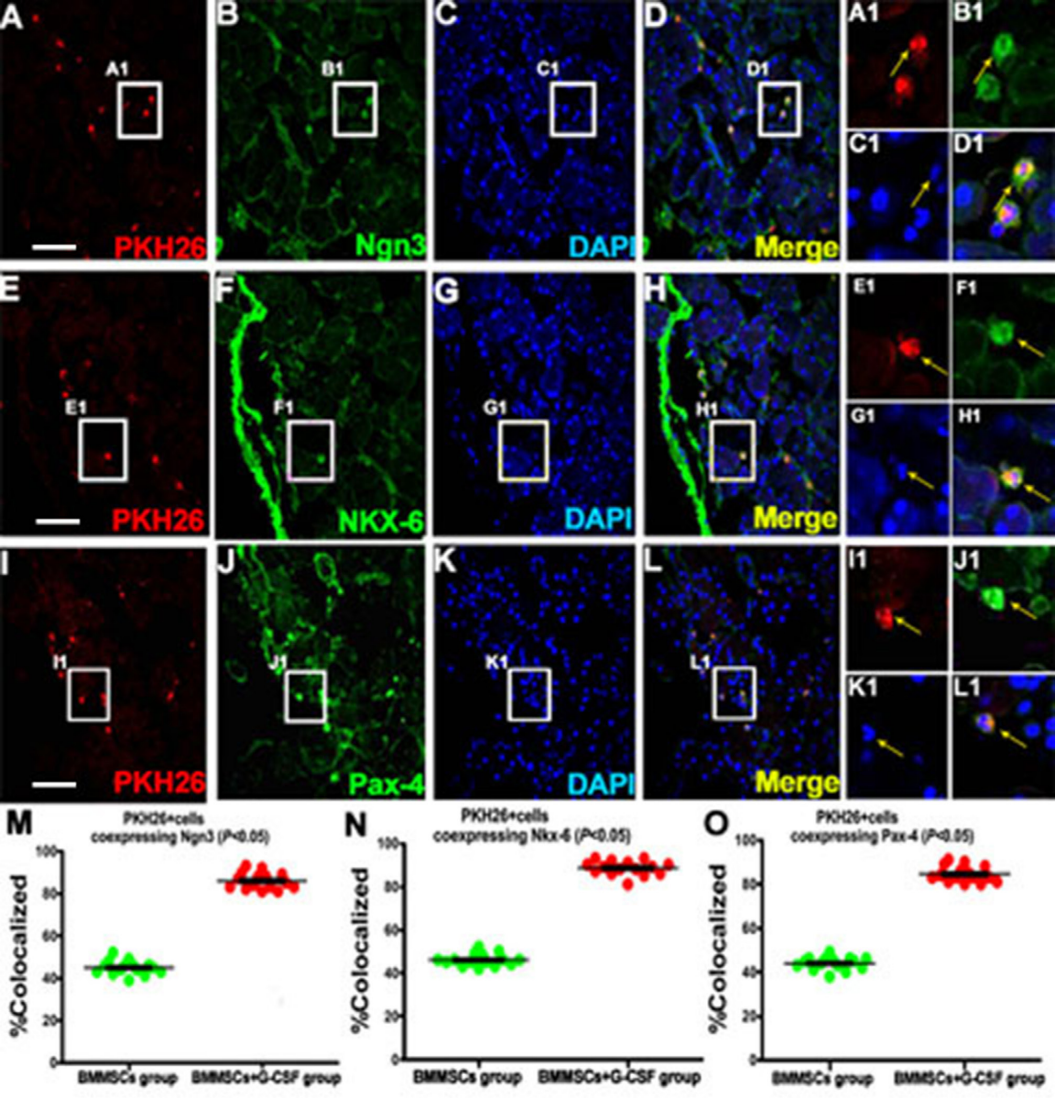
Fluorescent microscopic images showing potential *in situ* proliferation of transplanted BMMSCs in recipient rats with L-arginine–induced acute pancreatitis (**A, E, I**) The left panels show PHK26-positive BMMSCs in the pancreatic tissue in the BMMSC+G-CSF group at 3 weeks after transplantation. These cells show partial colocalization with the molecular markers of pancreatic stem cells or precursors, including (A, **B, C, D**) Ngn3, (E, **F, G, H**) Nkx-6 , and (I**, J, K, L**) Pax-4, in the BMMSC+G-CSF group. Colabeled cells are indicated by arrow. (**M, N, O**) Plots of the mean percentage ratios of PKH26-positive cells coexpressing Ngn3, Nkx- 6, and Pax-4 relative to the total population of PKH26-positive cells in individual animals with AP survived for 4 weeks after BMMSC transplantation and G-CSF administration. Scale bar = 100 μm for low magnification images, equivalent to 25 μm for enlarged inserts.

A panel of pancreatic cellular markers was used to explore *in situ* differentiation of the transplanted BMMSCs in recipient rats, including Nkx6 (NK6 homeobox 1), Ngn3 (neurogenin 3) and Pax4 (paired box 4) (Figure 4). In animals surviving 3 weeks after BMMSC transplantation and G-CSF administration, PKH26+ cells were colabeled with Ngn3 (Figure 4A–4D) at a rate of 45% ± 8.0% in the BMMSC group and 86% ± 6.0% in the BMMSC+G-CSF group (Figure 4M). In the control group and G-CSF group, there were no fluorescent cells (data not shown). The rate of Nkx-6 co-expression was 45% ± 7.0% in the BMMSC group and 89% ± 5.0% in the BMMSC+G-CSF group (Figure 4E–4H, 4N). The rate of Pax-4 co-expression was 44% ± 8.0% in the BMMSC group and 84% ± 6.0% in the BMMSC+G-CSF group (Figure 4I–4L, 4O).

### Analysis of proliferative mRNA expression in the pancreas of recipient rats

**Figure 5:**
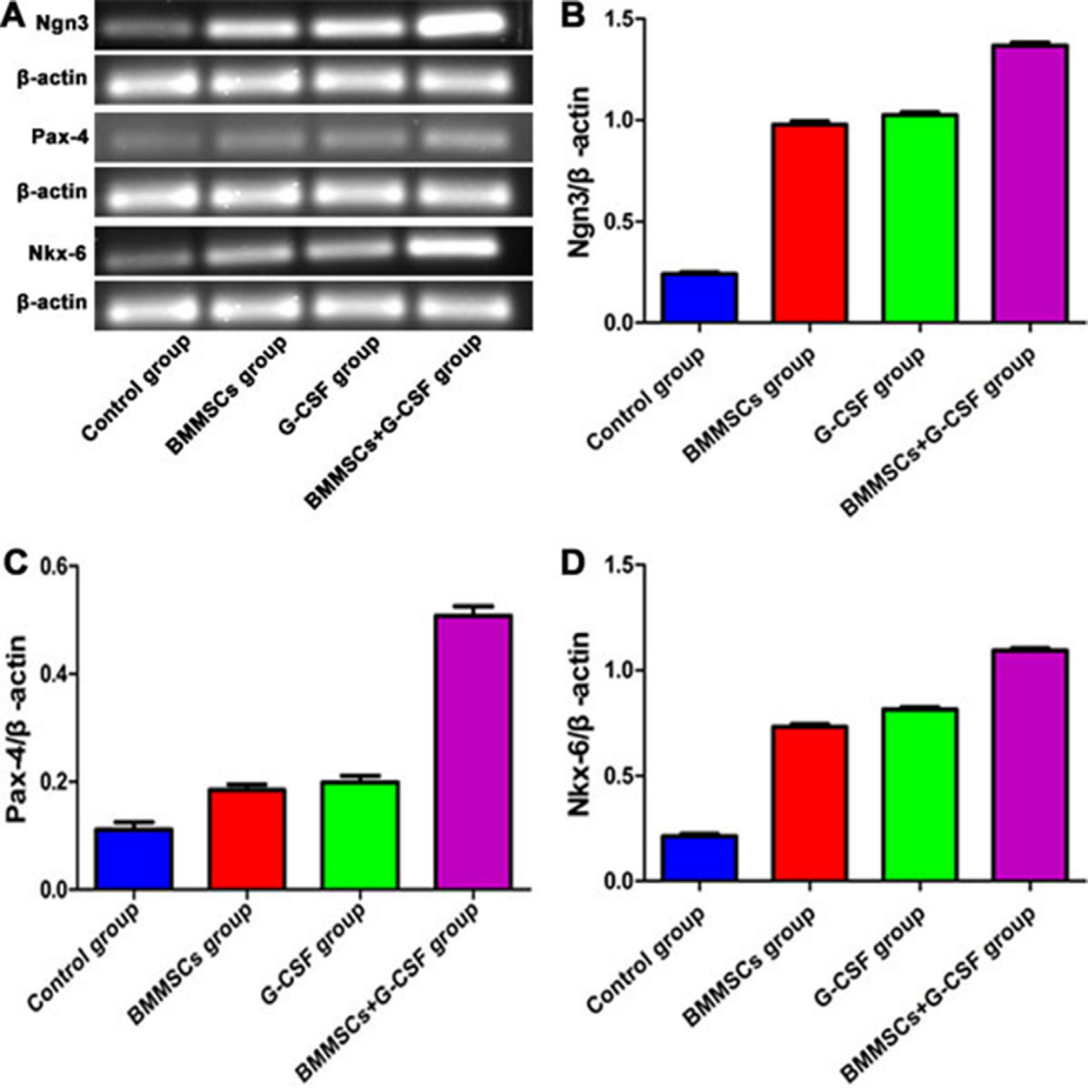
Effect of BMMSC transplantation and G-SCF therapy on pancreatic mRNA expression following L-arginine–induced injury in rats (**A**) The gene expression levels of Nkx-6, Ngn3, and Pax-4 were determined using RT-PCR analysis at 3 weeks after BMMSC and G-SCF administration. (**B**) Ngn3 expression was higher in the BMMSC+G-SCF group than in the BMMSC group and G-CSF group (*P* < 0.05 *vs*. the control group). (**C**) The grey value ratio between Pax-4 and β-actin was 0.51 in the BMMSC+G-CSF group, as compared to 0.11 in the control group, 0.18 in the BMMSC group, and 0.192 in the G-CSF group (*P* < 0.05). (**D**) The grey value ratio between Nkx-6 and β-actin was 1.10 in the BMMSC+G-CSF group, compared to 0.21 in the control group, 0.73 in the BMMSC group, and 0.82 in the G-CSF group (*P* < 0.05).

The mRNA levels of proliferative markers were determined using RT-PCR in the pancreatic tissues at 3 weeks after transplantation. The grey value ratio between Ngn3 and β-actin was 1.38 in the BMMSC+G-CSF group, compared to 0.24 in the control group, 0.99 in the BMMSC group and 1.02 in the G-CSF group. The Ngn3 expression in the treatment groups was higher than that in the control group, with the highest level in the BMMSC+G-CSF group (*P* < 0.05; Figure 5A, 5B). Similarly, there was a statistically significant increase in the expression levels of Pax-4 and Nkx-6 in each group after transplantation treatment (*P* < 0.05; Figure 5A, 5C, 5D).

### Improvement in serological and histological parameters after transplantation

**Figure 6:**
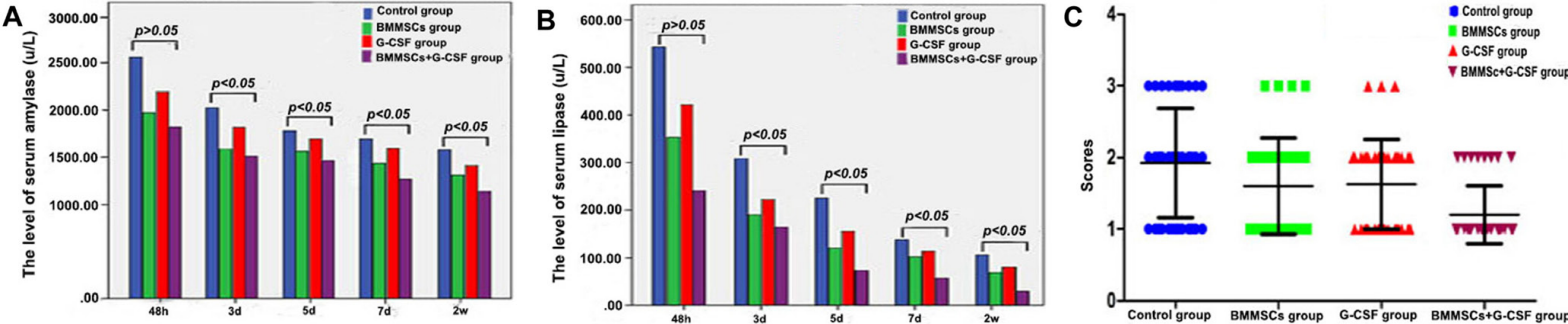
Improvement in blood amylase and serum lipase and overall histological parameters after transplantation There was no significant difference in serum amylase and serum lipase between the different groups at 2 d (*P* > 0.05). (**A, B**) Compared to the control group, the treatment groups showed obviously decreased serum amylase and serum lipase levels at 3 d, 5 d, 7 d, and 2 wk after transplantation, and the levels were lowest in BMMSC+G-SCF group (*P* < 0.05). (**C**) The macroscopic evaluation score was significantly lower in the BMMSC+G-SCF group than in the other two treatment groups (*P* < 0.05).

We withdrew blood samples from the eyeball in each group (*n* = 3) at different time points (2 d, 3 d, 5 d, 7 d, 2 wk), and determined serological changes in serum amylase and lipase. The data showed that there was no significant difference between the different groups at 2 d (*P* > 0.05). However, compared to the control group, the treatment groups showed significantly reduced serum amylase and lipase levels at 3 d, 5 d, 7 d, and 2 wk, with the levels in the BMMSC+G-CSF group being the lowest (*P* < 0.05; Figure 6A, 6B).

To determine whether the transplantation treatment was beneficial for pancreatic and overall histological recovery, we used the scoring system as established by Wacke et al. [28] to report histological integrity of the pancreas. The score of the macroscopic evaluation in the BMMSC+G-CSF group was significantly lower than that in the other two treatment groups (*P* < 0.05; Figure 6C).

## DISCUSSION

BMMSCs have potential therapeutic value in repairing tissues or organs in disease conditions. Research has suggested that BMMSC therapy facilitates tissue regeneration and improves functional recovery in a number of central and peripheral systems after acute or chronic injury [18–24, 37–38]. BMMSC transplantation has been considered for the treatment of pancreatitis. This treatment appears to promote the formation of pancreatic stellate cells [24]. Some studies show that transplanted BMMSCs may develop into pancreatic cells *in situ* [13–17]. Moreover, transplanted BMMSCs can reduce inflammation via downregulation of cytokines in experimental model of AP [17].

Consistent with literature [21, 25], rats treated with l-arginine show the gross anatomical appearance and histological changes of AP in the present study, including inflammatory cell infiltration, acinar necrosis, vacuolization and cavitation. To explore the therapeutic potential of BMMSC transplantation in our experimental AP model, BMMSCs were isolated from adult SD rats and expanded *in vitro*. The hematopoietic nature of the cultured cells was confirmed with a panel of specific membrane markers of BMMSCs, including CD29, CD34, CD44, CD45 and CD90 [22]. The *in vitro* expanded BMMSCs were efficiently labeled with PKH26 in order to track these cells *in vivo* for a few weeks. Following transplantation, the tagged BMMSCs migrate into the damaged tissue of the pancreas in the rat AP model, mostly present in the acini and ducts. These cells were colocalized with Pax-4. Thus, they appear to home in but proliferate *in situ* in the injured pancreatic tissue [13–17, 25, 26].

The administration of G-CSF is known to mobilize hematopoietic stem cells from the bone marrow into the peripheral blood [27–30]. This soluble factor can mobilize BMMSCs to home in tissue following myocardial or craniocerebral injuries and promote their differentiation towards target tissue cells [19, 20]. Likewise, G-CSF can enhance the proliferation of hepatic progenitor cells in both rodents and humans [31, 32]. G-CSF has been widely used in the treatment of leukemia [33]. The G-CSF receptor is a membrane protein, and Wexler et al. have proved that G-CSF promotes the proliferation of BMMSCs because these cells have G-CSF receptors [34]. G-CSF can alter cytokine expression in the liver, so hepatic stem cells proliferate under the influence of G-CSF [35]. Together, these experiments provide an important theoretical basis for the clinical treatment of AP with G-CSF for the mobilization of stem cells. In our previous experimental study, G-CSF was found to promote BMMSC homing to acutely injured livers in mice [36]. We further demonstrate here that G-CSF could promote BMMSC homing to the acutely injured pancreas to participate in pancreatic regeneration.

It is worth noting that in animals that have not infused with PKH26-tagged BMMSCs, no any fluorescently labeled cells can be found in tissue sections. The overall retention of transplanted BMMSCs in the pancreatic tissue, as assessed microscopically, appears not abundant. Given that the colabled cells are small in number, it is likely that the beneficial effect of BMMSCs may also involve potential soluble factors from the transplantation. This is consistent with the finding that addition of G-CSF improves the overall effect of the stem cell therapy in histological, biochemical and serological perspectives. Therefore, our experiment provides a theoretical basis for utilizing BMMSC transplantation and G-CSF administration in the clinical treatment of AP.

## MATERIALS AND METHODS

### Animals and main reagents

Male Sprague–Dawley (SD) rats weighing ∼200 g each were used for model preparation, while those weighing 80–100 g were used for extraction of BMMSCs. The animals were obtained from the animal facility of the Second Affiliated Hospital of Harbin Medical University, housed at constant temperature and humidity, with a 12/12 h light/dark illumination cycle and free access to food chow and water. All experimental procedures were approved by the Harbin Medical University Administrative Panel on Laboratory Animal Care. The reagents and medications used are shown below (Table 1).

**Table 1:** Primary antibodies and main reagents used in the present study

Antibody	Source	Product #	Dilution
PE anti-rat CD34	Santa Cruz	SC-7324	1:200
FITC anti-rat CD29	Biolegend	555005	1:100
APC anti-rat CD90	Biolegend	561409	1:100
FITC anti-rat CD44	Biolegend	550947	1:100
PE anti-rat CD45	Biolegend	554878	1:100
Pax-4 (rabbit IgG)	Santa Cruz	sc-98942	1:200
Nkx-6 (goat IgG)	Santa Cruz	sc-15030	1:200
Neurogenin3 (goat IgG)	Santa Cruz	Sc-23832	1:200

PE, phycoerythrin; FITC, fluoroisothiocyanate; APC, allophycocyanin.

### AP model preparation and grouping

**Figure 7:**
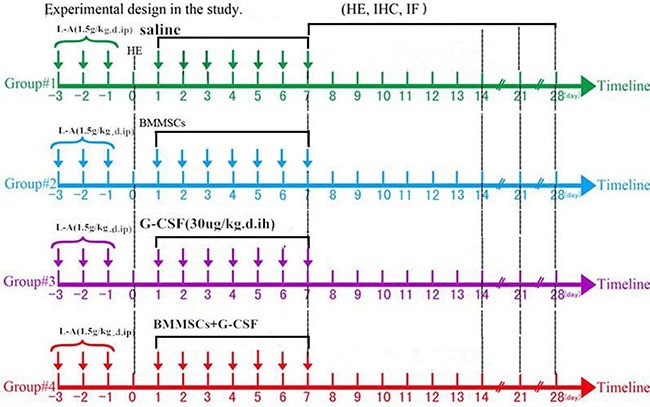
Experimental design L-Arginine was administrated on days –3 to –1, to animals, and pancreatic tissue was removed and examined visually and using hematoxylin and eosin (H&E) staining on day 0. In the next week (days 1–7), the rats in the different groups were intravenously injected with saline, BMMSCs, G-CSF, or BMMSC+G-CSF through tail vein. Histological, RT-PCR, and serological analyses were carried out from samples from animals surviving for 7, 14, 21, and 28 days.

The experimental model of AP was established via l-arginine administration in adult rats [21]. Animals were fasted for 12 h (allowed to drink) before model preparation, and then intraperitoneally injected with l-arginine (1.5 g/kg in 0.9% normal saline) two times a day, at 1 h interval, for 3 consecutive days. Four l-arginine treated rats were killed after being anesthetized with pentobarbital (50 mg/kg, i.p.) to evaluate the establishment of the model. The remaining animals (*n* = 60) were divided into four groups: (1) BMMSC transplantation and G-CSF treatment (BMMSC+G-CSF group, *n* = 15); (2) BMMSC transplantation only (BMMSC group, *n* = 15) and (3) G-CSF treatment only (G-CSF group, *n* = 15); and (4) vehicle (saline) infusion (control group, *n* = 15). Histological, serological and functional data were obtained following animal survival to different time points (Figure 7).

### BMMSC isolation, cultivation, and fluorescent pre-labeling

BMMSC isolation, cultivation and fluorescent pre-labeling were carried out as described previously [24]. Briefly, the femoral bones were removed from anesthetized (sodium pentobarbital, 50 mg/kg, i.p.) animals (*n* = 4). The medulla ossium was bathed twice with phosphate-buffered saline (PBS); the bone marrow was aspirated and suspended in a sterile centrifuge tube, centrifuged at 1500 rpm for 5 min at 25°C. The supernatant was discarded, and the cells were suspended in complete culture solution, and transferred into a sterile culture bottle. The cultured cells were pre-labeled with the red fluorescent lipophilic dye PKH26 (Sigma-Aldrich, St. Louis, US) [37]. After being blended with 15% complete culture solution and centrifuged at 1500 rpm for 2 min at 25°C, the PKH26-labeled cell suspension was adjusted to a concentration of approximately 3 × 10^7^ cells/mL. Subsequently, the cells with viability greater than 95%, as measured using trypan blue exclusion, were used for cell transplantation.

### BMMSC identification by fluorescence-activated cell sorting analysis

Fluorescence-activated cell sorting (FACS) was used to determine the expression of signature antigen markers of BMMSCs. Approximately 1 × 10^7^ cells were incubated in 2% fetal bovine serum in 0.01 M PBS (pH = 7.2) at 4°C for 20 min, and then supplemented with 1.0 μL of monoclonal antibodies to CD29, CD34, CD44, CD45 and CD90, with negative control incubated in fetal bovine serum without primary antibodies. The immunofluorescent signal was monitored and evaluated using the FACS Calibur with CellQuest software (Becton Dickinson, Franklin Lakes, NJ, USA).

### BMMSC transplantation and G-SCF administration

BMMSC transplantation was carried out via caudal vein injection [22, 38]. Thus, a total of 1.0 mL of the stem cell suspension (0.9% saline solution suspension containing 1 × 10^7^ cells), or 0.9% normal saline, were injected into a tail vein of a recipient rat at a constant slow speed. G-CSF was administered for 5 days via subcutaneous injection at a dosage of 30 μg/kg/ day.

### Pancreatic tissue preparation

Animals were perfused with PBS (pH = 7.4) via the ascending aorta after receiving an overdose of sodium pentobarbital (100 mg/kg, i.p.). The entire pancreas was removed from the abdominal cavity for visual inspection and tissue sampling. The degree of pancreatic injury was scored according to the method described by Wacke et al. [26]. Thus, score + (1) was defined if the pancreas was edematous, swollen, but no visible necrotic areas, calcification or discoloration; score ++ (2) if it was edematous with small solitary necrotic areas and a small number of fine points of calcification; and score +++ (3) if patches of calcification and necrosis were seen on the pancreas as extensive grayish-white areas.

Each pancreas was then divided into three parts. One part was rinsed in cold PBS several times and then immersed in 4% paraformaldehyde for 24 h. This sample was embedded in paraffin, sectioned at a thickness of 3 μm, and used for histology and immunohistochemistry. The second part was also fixed in 4% paraformaldehyde, treated with 10%, 20% and 30% sucrose (every 6 h), sectioned in a cryostat at 4 μm and used for immunofluorescence. The last part was snap-frozen in liquid nitrogen for polymerase chain reaction (PCR) assays. Blood was collected from the rat eyeball and centrifuged at 2000 rpm for 10 min (4°C), with the resultant serum used for biochemical studies.

### Histological and immunohistochemical processing and image analysis

Pancreatic sections were stained with hematoxylin and eosin (H.E.). For immunofluorescence, dewaxed or cryostat sections were preincubated for 30 min in PBS containing 5% normal donkey serum, followed by incubations with rabbit anti-Pax-5, goat anti-Nkx-6 or goat anti-Ngn3 antibody, at 4°C overnight. The sections were washed with PBS, and then, incubated with Alexa Fluor^®^ 488 conjugated goat anti-rabbit IgG or rabbit anti-goat IgG (1:200, Invitrogen, USA) for 2 h. The sections were counterstained with DAPI (4′,6-diamidino-2-phenylindole), and mounted with an anti-fading medium before microscopic examination.

Cells with and without colabeling were counted in confocal immunofluorescent images. For each type of double labeling, at least 10 sets of images were obtained from the laminar region of each pancreas tissue sample at 10×. At least 200 cells were analyzed to determine the rate of colocalization between two given markers in each animal.

### Reverse transcription PCR

RNA was extracted using the TRIzol reagent (Sangon Biotech, Shanghai) according to the manufacturer's instructions and stored at −70°C until use. Next, cDNAs were synthesized by reverse transcription (RT) using a commercial kit (Sangon Biotech, Shanghai). The following forward (F) and reverse (R) primers were used for RT-PCR amplifications.

Nkx-6 (293 bp), 5′-AAGGGGACTTCGGAGAA TGA-3′ (F) and 5′-CCGCCACAATTTCTAGGTTAA-3′ (R); Pax-4 (293 bp), 5′-GCCCTCTGCTCCTGAGTGAA-3′ (F) and 5′-GCCAACTGGCAAACTGAAAA-3′ (R); Ngn3 (170 bp), 5′-CACTCAGCAAACAGCGAAGA-3′ (F) and 5′-GATGTAGTTGTGGGCGAAGC-3′ (R); and rat β-actin (157 bp), 5′-TTTTCCAGCCTTCCTTCTTG-3′ (F) and 5′-CAATGCCTGGGTACATGGTGGTACC-3′ (R).

### Statistical analysis

Data were expressed as mean ± SEM. Statistical analyses were performed using two-way analysis of variance (ANOVA) for repeated measures (Prism GraphPad 4.1, San Diego, CA). *P* < 0.05 was considered statistically significant. Figures were assembled with Photoshop 7.1 and saved as TIFF files.

## CONCLUSIONS

The present study shows that *in vitro* expanded BMMSCs may relocate to and proliferate locally in injured pancreas in a rat model of acute pancreatitis. While BMMSC transplantation alone shows a certain degree of therapeutic effect on tissue repair, co-administration of G-CSF has a synergetic benefit by improving the histopathological, serological and functional recovery of pancreas following the experimental injury.

## Abbreviations

AP: Acute pancreatitis
BMMSCs: Bone marrow mesenchymal stem cells
G-CSF: granulocyte colony-stimulating factor
MSCs: mesenchymal stem cells
PBS: phosphate-buffered saline
FACS: fluorescence-activated cell sorting
